# Supplementary material for: Analysis of the factors influencing the proximity and agreement between critical power and maximal lactate steady state: a systematic review and meta-analyses
Source: PeerJ. 2025 Mar 18;13:e19060. doi: 10.7717/peerj.19060 (PMC11927562; doi:10.7717/peerj.19060)
Supplement: Supplemental Information 2 — Keywords search strategy performed using different combinations created through the utilization of the Boolean operators (i.e., AND and OR). [file peerj-13-19060-s002.pdf]

Keyword search strategy on Pubmed, Scopus, and Web of Science

(("critical power" OR "maximal lactate steady state" OR "maximal metabolic steady state") AND ("respiratory compensation point" OR threshold)) OR ("critical power" AND "maximal lactate steady state") OR ("critical power" AND "maximal metabolic steady state") OR ("maximal lactate steady state" AND "maximal metabolic steady state")
