# Supplementary material for: Analysis of the factors influencing the proximity and agreement between critical power and maximal lactate steady state: a systematic review and meta-analyses
Source: PeerJ. 2025 Mar 18;13:e19060. doi: 10.7717/peerj.19060 (PMC11927562; doi:10.7717/peerj.19060)
Supplement: Supplemental Information 3 [file peerj-13-19060-s003.docx]

*Meta-analyses of the mean differences in the studies including both PO and* $\dot{V̇}$O_2_

The pooled MD in PO between CP and MLSS was 10.59 W (95% CI: −4.34 to 25.51, SE = 5.375, t = 1.969, PI = −24.15 to 45.32), these differences indicate that, on average, CP is not different from MLSS (p = 0.120). Please, refer to the forest plot (Fig. 1S) for a visual representation of both the pooled MD and CI, and non-pooled MD and CI for each study included in the meta-analysis. The MD expressed in W showed a significant heterogeneity (Q_(4)_ = 51.364, τ = 11.296, p < 0.001).

INSERT **Fig. 1S** APPROXIMATELY HERE

In terms of $\dot{V̇}$O_2_, the pooled MD between CP and MLSS was 0.11 L**·**min^-1^ (95% CI: −0.04 to 0.25, SE = 0.052, t = 2.017, PI = −0.21 to 0.42). These differences indicate that, on average, CP and MLSS are not different (p = 0.114) when expressed as $\dot{V̇}$O_2_. The forest plot in Fig.2S provides a visual representation of both the pooled MD and CI, and non-pooled MD and CI for each study included in the meta-analysis. The MD, expressed in L**·**min^-1^, showed a significant heterogeneity (Q_(4)_ = 14.615, τ = 0.099, p = 0.006).

INSERT **Fig. 2S** APPROXIMATELY HERE
