# Supplementary material for: Analysis of the factors influencing the proximity and agreement between critical power and maximal lactate steady state: a systematic review and meta-analyses
Source: PeerJ. 2025 Mar 18;13:e19060. doi: 10.7717/peerj.19060 (PMC11927562; doi:10.7717/peerj.19060)
Supplement: Supplemental Information 4 — SMD, standardized mean difference; SMDPO, standardized mean difference in terms of PO; SMD .VO2, standardized mean difference in terms of O2; D SMDPO .VO2 , difference between SDMs computed as SMD_PO minus SMD .VO2; CP, critical power; MLSS, maximal lactate steady state. [file peerj-13-19060-s004.docx]

| **Descriptive results of the standardized mean differences (SMDs), computed as the difference between CP and MLSS, in PO and** $\dot{\boldsymbol{V}\mathbf{̇}}$**O_2_, and their differences.** | | | |
| --- | --- | --- | --- |
| *Authors* | SMD_PO_ | SMD*_V̇_*_O2_ | ΔSMD_PO−_*_V̇_*_O2_ |
| Keir et al., 2015 | 0.15 | 0.11 | 0.04 |
| Ozkaya et al., 2022 | 2.52 | 1.41 | 1.11 |
| Okuno et al., 2011 | 0.54 | 0.18 | 0.36 |
| Caen et al., 2022 a) | 0.46 | 0.55 | −0.09 |
| Caen et al., 2022 b) | 0.39 | 0.55 | −0.16 |
| SMD, standardized mean difference; SMD_PO_, standardized mean difference in terms of PO; SMD*_V̇_*_O2_, standardized mean difference in terms of $\dot{V̇}$O_2_; ΔSMD_PO−_*_V̇_*_O2_, difference between SDMs computed as SMD_PO_ minus SMD*_V̇_*_O2_; CP, critical power; MLSS, maximal lactate steady state. | | | |
