# Supplementary material for: Analysis of the factors influencing the proximity and agreement between critical power and maximal lactate steady state: a systematic review and meta-analyses
Source: PeerJ. 2025 Mar 18;13:e19060. doi: 10.7717/peerj.19060 (PMC11927562; doi:10.7717/peerj.19060)
Supplement: Supplemental Information 5 [file peerj-13-19060-s005.docx]

This article is intended for individuals and exercise professionals involved in aerobic exercise testing and prescription, including researchers, exercise physiologists, healthcare professionals, sports scientists, and coaches. It provides insights into the relationship between critical power and maximal lactate steady state, as well as the broader context of identifying aerobic exercise thresholds and their applications in exercise testing and prescription.
