# Supplementary figures and images for: Analysis of the factors influencing the proximity and agreement between critical power and maximal lactate steady state: a systematic review and meta-analyses
Source: PeerJ. 2025 Mar 18;13:e19060. doi: 10.7717/peerj.19060 (PMC11927562; doi:10.7717/peerj.19060)

**Study****Weight MD [95% CI]**

Keir et al., 2015

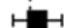

20.70% 2.00 [-4.79, 8.79]

Caen et al., 2022a

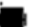

22.03% 3.00 [-0.72, 6.72]

Caen et al., 2022b

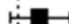

19.85% 6.00 [-2.31, 14.31]

Okuno et al., 2011

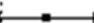

16.71% 12.60 [-0.60, 25.80]

Ozkaya et al., 2022

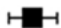

20.72% 30.00 [23.24, 36.76]

RE Model

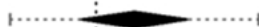

100% 10.59 [-4.34, 25.51]

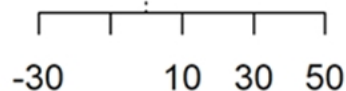

Supplement: Supplemental Information 6 [file peerj-13-19060-s006.pdf]

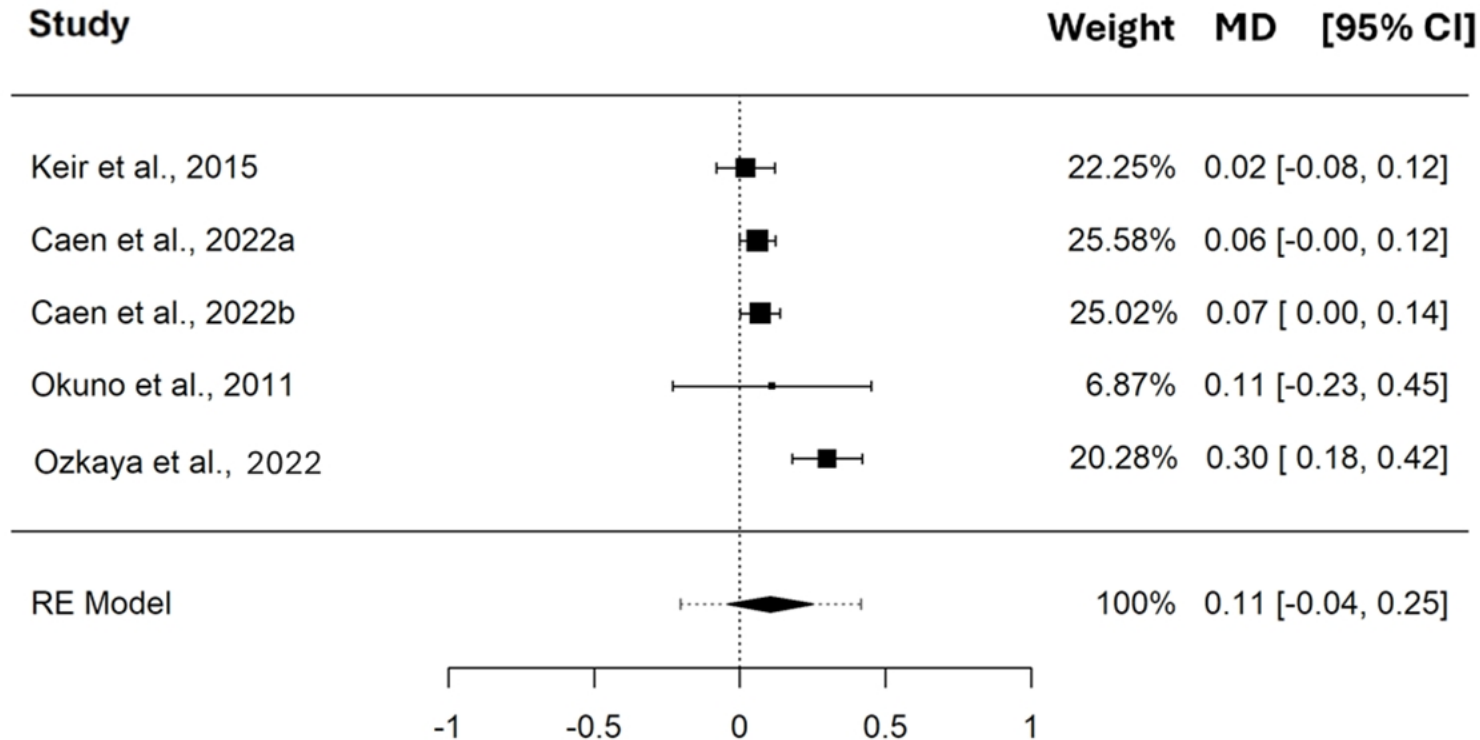

Supplement: Supplemental Information 7 [file peerj-13-19060-s007.pdf]
